# Supplementary material for: Evaluating the Capability of Epipremnum aureum and Its Associated Phylloplane Microbiome to Capture Indoor Particulate Matter Bound Lead
Source: Plants (Basel). 2025 Sep 23;14(19):2956. doi: 10.3390/plants14192956 (PMC12525747; doi:10.3390/plants14192956)
Supplement: Supplementary file 1 [file plants-14-02956-s001.zip › plants-3874152-supplementary.pdf]

Evaluating the capability of *Epipremnum aureum* and its associated phylloplane microbiome to capture indoor particulate matter bound lead

Diego G. Much[a,b*]; Anabel Saran[a,b*]; Luciano J. Merini[a,c]; Jaco Vangronsveld[c,d]; Sofie Thijs[c]

[a] Agencia de Investigación Científica (AIC) sede Santa Rosa, Corona Martínez 430, Santa Rosa, La Pampa, 6300, Argentina.

[b] Consejo Nacional de Investigaciones Científicas y Técnicas (CONICET), Godoy Cruz 2290, CABA, 1425, Argentina

[c] Environmental Biology, Centre for Environmental Sciences, Hasselt University, Diepenbeek, Belgium.

[d] Department of Plant Physiology and Biophysics, Institute of Biological Sciences, Maria Curie Sklodowska University, Lublin, Poland.

*Corresponding authors: Diego G. Much, e-mail: diego.much@live.com.ar; Anabel Saran, e-mail: saran.anabel@gmail.com

Table S1. Temporal variation of $PM_{2.5}$, PM_{10}, total particles, carbon dioxide (CO_2) and formaldehyde (HCHO) concentrations during three shoots in the shooting room, measured on the same day using a Temtop M2000 portable air quality meter.

shoot	T (s)	$PM_{2.5}$ (μg · m^{-3})	PM_{10} (μg · m^{-3})	Particles (count · L)	CO_2 (ppm)	HCHO (mg · m^{-3})
First	0	9.1	12.8	3181	754	0.168
	10	8.9	12.7	3127	753	0.168
	20	15.8	23.7	6391	753	0.168
	30	32.9	50.6	9447	754	0.167
	40	36.9	56.5	9458	755	0.167
	50	34.4	52.8	7897	759	0.167
	60	30.2	47.4	6213	761	0.167
	70	27.5	42.5	5360	764	0.167
	80	24.8	37	4339	769	0.168
	90	23.1	33.7	3995	775	0.168
	100	17.1	24.6	4114	785	0.168
Second	0	12.06	18.1	4017	849	0.166
	10	12.4	17.7	4260	850	0.166
	20	12.5	19	5909	849	0.165
	30	12.7	19.5	7449	848	0.164
	40	14.6	21.7	7261	846	0.164
	50	19.7	28.4	5884	846	0.163
	60	20.01	28.7	5183	848	0.163
	70	20.01	29.3	5229	852	0.164
	80	18.7	27.9	4981	856	0.165
	90	16.7	25.3	4642	861	0.165
	100	16.1	24.5	4273	863	0.165
Third	0	15	22.7	4855	877	0.164
	10	15.2	23.4	5419	878	0.163
	20	23.1	34.9	7042	879	0.163
	30	21.4	32.4	5762	880	0.163
	40	19	28.8	5053	880	0.162
	50	17.7	26.3	4717	882	0.162
	60	17.2	26.2	5176	884	0.163
	70	18.1	28.6	5775	887	0.164
	80	19.3	30	5501	896	0.163
	90	19.5	29.5	5343	901	0.163
	100	20	29.6	5174	904	0.163

Table S2. Phylloplane PM_{10}, $PM_{2.5}$, and black carbon (BC) load (n = 5) are presented with standard deviation values for reference (RR) and shooting (SR) rooms at different times of exposure. Tests used to compare variables between groups at the same time point and their *P*-values are provided.

**Variable**		**Site**		**Test**	***p*-value**
		**RR**	**SR**		
**PM_{10} ($mg \cdot cm^{-2}$)**	T_0	$1.13e^{-04} \pm 6.20e^{-06}$	$7.06e^{-05} \pm 2.39e^{-05}$	T-test	0.0148 *
	T_3	$2.61e^{-04} \pm 8.69e^{-05}$	$5.26e^{-04} \pm 3.55e^{-05}$	Mann-Whitney U	0.0317 *
	T_6	$2.87e^{-04} \pm 9.65e^{-05}$	$3.23e^{-04} \pm 7.86e^{-05}$	T-test	0.5267
	T_{12}	$3.20e^{-04} \pm 1.32e^{-05}$	$3.18e^{-04} \pm 1.26e^{-05}$	T-test	0.7465
**$PM_{2.5}$ ($mg \cdot cm^{-2}$)**	T_0	$9.39e^{-05} \pm 2.76e^{-05}$	$8.42e^{-05} \pm 2.00e^{-05}$	T-test	0.5435
	T_3	$2.79e^{-04} \pm 3.54e^{-05}$	$4.89e^{-04} \pm 1.03e^{-05}$	T-test	0.0270 *
	T_6	$2.85e^{-04} \pm 6.59e^{-05}$	$3.70e^{-04} \pm 8.34e^{-05}$	T-test	0.1131
	T_{12}	$2.45e^{-04} \pm 1.94e^{-05}$	$2.28e^{-04} \pm 1.55e^{-05}$	Mann-Whitney U	0.2933
**BC load (no. particles · mL^{-1})**	T_0	$2.89e^{05} \pm 1.08e^{05}$	$2.58e^{05} \pm 1.52e^{05}$	Mann-Whitney U	0.5628
	T_{12}	$7.26e^{05} \pm 1.83e^{05}$	$1.94e^{06} \pm 8.81e^{05}$	Mann-Whitney U	$3.28e^{-05}$ ****

* and **** denotes significantly different at $p < 0.05$ and $p < 0.0001$, respectively.

Table S3. Summary of Statistical Results for Metal and Metalloid Concentrations in *Epipremnum aureum* Leaves.

**Element**	**Between sites**				
	**Time**	**Comparison**	**Test**	**p-value**	**Significant**
Ca	T_0	RR vs. SR	Mann-Whitney U	0.4206	No
Ca	T_3	RR vs. SR	Mann-Whitney U	0.0952	No
Ca	T_6	RR vs. SR	Mann-Whitney U	0.5476	No
Ca	T_{12}	RR vs. SR	Mann-Whitney U	0.0159	Yes
Cd	T_0	RR vs. SR	Mann-Whitney U	0.0254	Yes
Cd	T_3	RR vs. SR	Mann-Whitney U	0.1508	No
Cd	T_6	RR vs. SR	Mann-Whitney U	-	-
Cd	T_{12}	RR vs. SR	Mann-Whitney U	0.0079	Yes
Cu	T_0	RR vs. SR	Mann-Whitney U	0.5476	No
Cu	T_3	RR vs. SR	Mann-Whitney U	0.3095	No
Cu	T_6	RR vs. SR	Mann-Whitney U	0.4206	No
Cu	T_{12}	RR vs. SR	Mann-Whitney U	1.000	No
Fe	T_0	RR vs. SR	Mann-Whitney U	1.000	No
Fe	T_3	RR vs. SR	Mann-Whitney U	0.5476	No
Fe	T_6	RR vs. SR	Mann-Whitney U	0.6905	No
Fe	T_{12}	RR vs. SR	Mann-Whitney U	0.6905	No
K	T_0	RR vs. SR	T-test	0.7043	No
K	T_3	RR vs. SR	T-test	0.5278	No
K	T_6	RR vs. SR	T-test	0.1463	No
K	T_{12}	RR vs. SR	T-test	0.8448	No
Mg	T_0	RR vs. SR	T-test	0.5650	No
Mg	T_3	RR vs. SR	T-test	0.0434	Yes
Mg	T_6	RR vs. SR	T-test	0.7910	No
Mg	T_{12}	RR vs. SR	T-test	0.0041	Yes
Mn	T_0	RR vs. SR	T-test	0.7112	No
Mn	T_3	RR vs. SR	T-test	0.1246	No
Mn	T_6	RR vs. SR	T-test	0.4892	No
Mn	T_{12}	RR vs. SR	T-test	0.7425	No

Na	T_0	RR vs. SR	T-test	0.5645	No
Na	T_3	RR vs. SR	T-test	0.4173	No
Na	T_6	RR vs. SR	T-test	0.5831	No
Na	T_{12}	RR vs. SR	T-test	0.2024	No
P	T_0	RR vs. SR	T-test	0.4697	No
P	T_3	RR vs. SR	T-test	0.0092	Yes
P	T_6	RR vs. SR	T-test	0.5200	No
P	T_{12}	RR vs. SR	T-test	0.1872	No
S	T_0	RR vs. SR	T-test	0.4422	No
S	T_3	RR vs. SR	T-test	0.2892	No
S	T_6	RR vs. SR	T-test	0.5825	No
S	T_{12}	RR vs. SR	T-test	0.1555	No
Zn	T_0	RR vs. SR	Mann-Whitney U	0.2222	No
Zn	T_3	RR vs. SR	Mann-Whitney U	0.5476	No
Zn	T_6	RR vs. SR	Mann-Whitney U	0.5476	No
Zn	T_{12}	RR vs. SR	Mann-Whitney U	0.0434	Yes

	**Within the same site**				
**Element**	**Site**	**Comparison**	**Test**	**p-value**	**Significant**
Ca	RR	T_3 vs. T_0	Wilcoxon signed-rank	0.8125	No
Ca	RR	T_6 vs. T_3	Wilcoxon signed-rank	0.1250	No
Ca	RR	T_{12} vs. T_6	Wilcoxon signed-rank	0.1250	No
Ca	SR	T_3 vs. T_0	Wilcoxon signed-rank	0.4375	No
Ca	SR	T_6 vs. T_3	Wilcoxon signed-rank	1.000	No
Ca	SR	T_{12} vs. T_6	Wilcoxon signed-rank	0.0625	No
Cd	RR	T_3 vs. T_0	Wilcoxon signed-rank	0.1250	No
Cd	RR	T_6 vs. T_3	Wilcoxon signed-rank	0.0625	No
Cd	RR	T_{12} vs. T_6	Wilcoxon signed-rank	0.0625	No
Cd	SR	T_3 vs. T_0	Wilcoxon signed-rank	0.0625	No
Cd	SR	T_6 vs. T_3	Wilcoxon signed-rank	0.0625	No
Cd	SR	T_{12} vs. T_6	Wilcoxon signed-rank	0.0625	No
Cu	RR	T_3 vs. T_0	Wilcoxon signed-rank	0.6250	No
Cu	RR	T_6 vs. T_3	Wilcoxon signed-rank	0.0625	No

Cu	RR	T_{12} vs. T_6	Wilcoxon signed-rank	0.3125	No
Cu	SR	T_3 vs. T_0	Wilcoxon signed-rank	0.4375	No
Cu	SR	T_6 vs. T_3	Wilcoxon signed-rank	1.000	No
Cu	SR	T_{12} vs. T_6	Wilcoxon signed-rank	0.8125	No
Fe	RR	T_3 vs. T_0	Wilcoxon signed-rank	0.0625	No
Fe	RR	T_6 vs. T_3	Wilcoxon signed-rank	1.000	No
Fe	RR	T_{12} vs. T_6	Wilcoxon signed-rank	0.6250	No
Fe	SR	T_3 vs. T_0	Wilcoxon signed-rank	0.1875	No
Fe	SR	T_6 vs. T_3	Wilcoxon signed-rank	0.8125	No
Fe	SR	T_{12} vs. T_6	Wilcoxon signed-rank	1.000	No
K	RR	T_3 vs. T_0	Paired t-test	0.8071	No
K	RR	T_6 vs. T_3	Paired t-test	0.3731	No
K	RR	T_{12} vs. T_6	Paired t-test	0.8623	No
K	SR	T_3 vs. T_0	Paired t-test	0.5086	No
K	SR	T_6 vs. T_3	Paired t-test	0.4232	No
K	SR	T_{12} vs. T_6	Paired t-test	0.2670	No
Mg	RR	T_3 vs. T_0	Paired t-test	0.5701	No
Mg	RR	T_6 vs. T_3	Paired t-test	0.6592	No
Mg	RR	T_{12} vs. T_6	Paired t-test	0.3391	No
Mg	SR	T_3 vs. T_0	Paired t-test	0.1169	No
Mg	SR	T_6 vs. T_3	Paired t-test	0.3035	No
Mg	SR	T_{12} vs. T_6	Paired t-test	0.0040	Yes
Mn	RR	T_3 vs. T_0	Paired t-test	0.9807	No
Mn	RR	T_6 vs. T_3	Paired t-test	0.4558	No
Mn	RR	T_{12} vs. T_6	Paired t-test	0.0040	Yes
Mn	SR	T_3 vs. T_0	Paired t-test	0.2585	No
Mn	SR	T_6 vs. T_3	Paired t-test	0.3087	No
Mn	SR	T_{12} vs. T_6	Paired t-test	0.1403	No
Na	RR	T_3 vs. T_0	Paired t-test	0.5004	No
Na	RR	T_6 vs. T_3	Paired t-test	0.6326	No
Na	RR	T_{12} vs. T_6	Paired t-test	0.0785	No
Na	SR	T_3 vs. T_0	Paired t-test	0.5889	No
Na	SR	T_6 vs. T_3	Paired t-test	0.4886	No

Na	SR	T_{12} vs. T_6	Paired t-test	0.1068	No
P	RR	T_3 vs. T_0	Paired t-test	0.1164	No
P	RR	T_6 vs. T_3	Paired t-test	0.9220	No
P	RR	T_{12} vs. T_6	Paired t-test	0.1976	No
P	SR	T_3 vs. T_0	Paired t-test	0.0025	Yes
P	SR	T_6 vs. T_3	Paired t-test	0.2288	No
P	SR	T_{12} vs. T_6	Paired t-test	0.1918	No
S	RR	T_3 vs. T_0	Paired t-test	0.2628	No
S	RR	T_6 vs. T_3	Paired t-test	0.6423	No
S	RR	T_{12} vs. T_6	Paired t-test	0.2651	No
S	SR	T_3 vs. T_0	Paired t-test	0.1291	No
S	SR	T_6 vs. T_3	Paired t-test	0.3694	No
S	SR	T_{12} vs. T_6	Paired t-test	0.1288	No
Zn	RR	T_3 vs. T_0	Wilcoxon signed-rank	0.3125	No
Zn	RR	T_6 vs. T_3	Wilcoxon signed-rank	0.3125	No
Zn	RR	T_{12} vs. T_6	Wilcoxon signed-rank	0.1250	No
Zn	SR	T_3 vs. T_0	Wilcoxon signed-rank	0.6250	No
Zn	SR	T_6 vs. T_3	Wilcoxon signed-rank	0.4375	No
Zn	SR	T_{12} vs. T_6	Wilcoxon signed-rank	0.0425	Yes

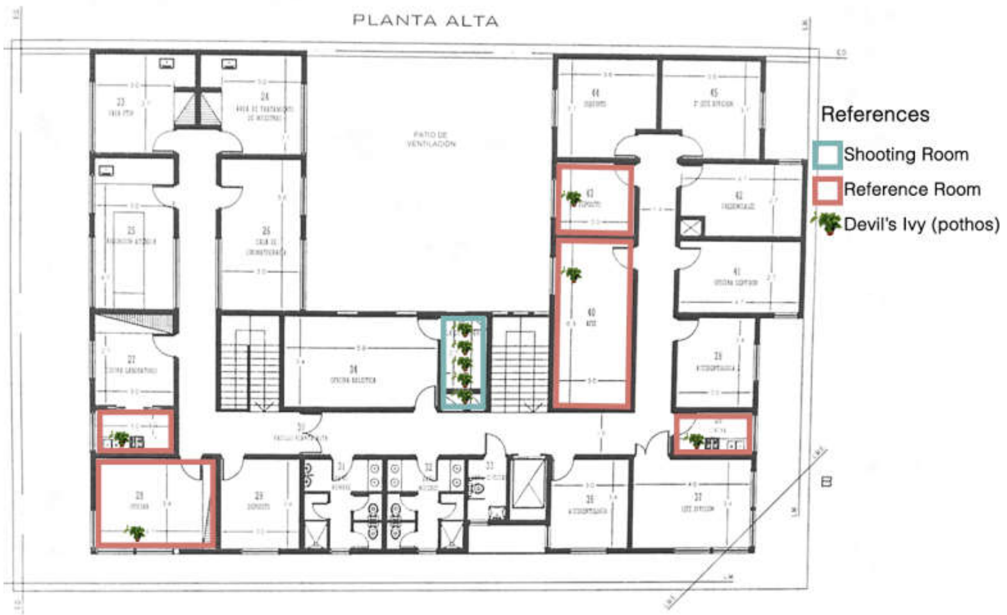

Figure S1. Floor plan of the reference rooms (RR) and shooting room (SR) housing the plants.

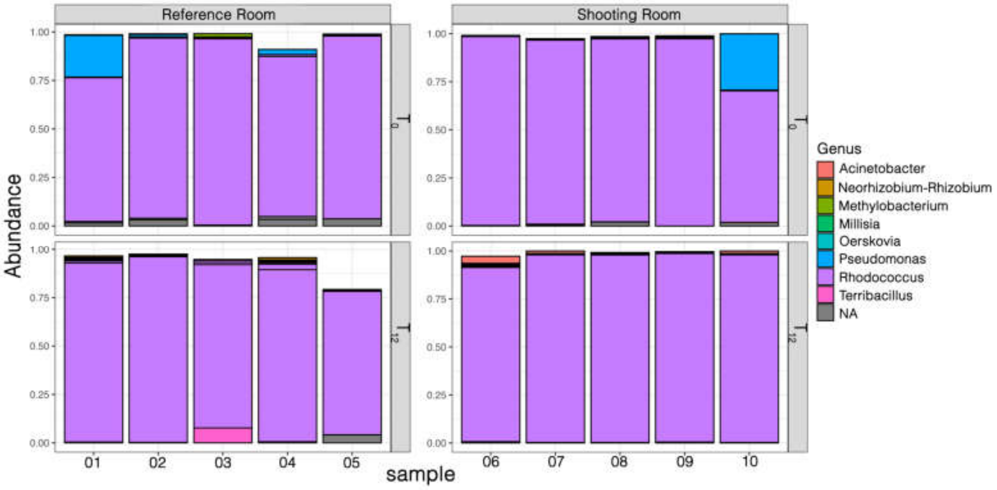

Figure S2. Relative abundances of taxa at the genus level of phylloplane samples from *E. Aureum* leaves located in the reference room (RR) and the shooting room (SR) at the initial time (T0) and after 12 months (T12).
